# Torque Teno Virus (TTV) Plasma Load and Immune Reconstitution Post-Transplantation in Patients with Lymphoproliferative Disorders: A Systematic Review

**DOI:** 10.3390/pathogens15010105

**Published:** 2026-01-19

**Authors:** Eugenia Quiros-Roldan, Martina Salvi, Maria Alberti, Giorgio Tiecco, Giorgio Biasiotto, Roberto Bresciani, Diego Bertoli, Alessandra Sottini, Maria Antonia De Francesco

**Affiliations:** 1 Unit of Infectious and Tropical Diseases, Department of Clinical and Experimental Sciences, University of Brescia and ASST Spedali Civili of Brescia, 25123 Brescia, Italy; eugeniaquiros@yahoo.it (E.Q.-R.); m.salvi026@unibs.it (M.S.); m.alberti035@studenti.unibs.it (M.A.); g.tiecco@unibs.it (G.T.); 2 Department of Molecular and Translational Medicine, University of Brescia, 25123 Brescia, Italy; giorgio.biasiotto@unibs.it (G.B.); roberto.bresciani@unibs.it (R.B.); 3 Highly Specialized Laboratory, ASST Spedali Civili of Brescia, 25123 Brescia, Italy; diego.bertoli@asst-spedalicivili.it (D.B.); alessandra.sottini@asst-spedalicivili.it (A.S.)

**Keywords:** TTV, HSCT, opportunistic infections, GVHD

## Abstract

Torque Teno Virus (TTV), a common and genetically diverse component of the human virome, is not linked to any known disease but reflects immune status. Its plasma viral load has shown clinical relevance in solid organ transplant recipients, correlating it with immunosuppression when present at high levels. However, the clinical significance of TTV viral load in hematopoietic stem cell transplantation (HSCT) recipients is less understood. This systematic review aims to evaluate whether plasma TTV DNA load directly correlates with the degree of T-cell immune reconstitution after HSCT, supporting its potential role as a biomarker for immune competence. The study protocol was registered in the PROSPERO International Prospective Register of Systematic Reviews (CRD420251116208) and followed the Preferred Reporting Items for Systematic Reviews and Meta-Analyses (PRISMA) guidelines. Twenty-one studies were included. The results showed concordant data about TTV kinetics with peak levels reaching approximately between +90 to +120 days after transplantation. Contradictory results have instead been found for the association of TTV load with immune parameters (lymphocyte counts, viral opportunistic infection, and development of acute graft versus host diseases). Even if a low-risk bias assessment was classified in most studies (67%), it was possible to identify important clinical and methodological differences between them, which might account for the different findings observed. Therefore, future larger studies with standardized protocols are needed to assess whether TTV viral load can serve as a reliable tool for guiding clinical decisions in the context of HSCT.

## 1. Introduction

Torque Teno Virus (TTV) is considered an important component of the human virome [1,2,3] and it was never associated with any pathology in humans [4]. The virus, characterized by a small negative-sense circular single-stranded DNA, belongs to the family *Anelloviridae*, which includes, to date, 31 genera and 155 species [5]. Human colonization is due to three genera of *Anelloviridae*: *Alphatorquetenovirus* (including TTV), *Betatorquetenovirus* (including Torque teno mini virus [TTMV]) and *Gammatorquetenovirus* (including Torque teno midi virus [TTMDV]) [6,7]. TTV exhibits a high genetic variability, which can be divided into five genetic groups differing by approximately 50% of their genomic sequence and different genotypes inside the groups (characterized by a difference of about 30% in their genomic sequence) [8]. Various studies show that TTV is acquired early in life by many transmission routes such as breast milk, saliva, and fecal–oral and respiratory transmission [9,10], reaching a prevalence ranging from 65% to 94% in older age [11]. The virus is prevalently localized in lymphocyte cells, even if it has been detected in different tissues and fluids, leading thereby to the definition of TTV as a “pantropic virus” [12,13]. Its replication is kept under control by a competent immune system. So far, high plasma TTV viral loads have been associated with immunosuppression, and low plasma TTV viral loads have been associated with an increased risk of organ rejection in subjects who have received solid organ transplants (SOTs) [14]. This peculiarity makes TTV a promising biomarker to monitor the immunological state of individuals who are immunosuppressed for various reasons.

While in recent years, many studies have shown the clinical value of TTV viral load evaluation in SOT population, there is limited evidence for this in hematopoietic stem cell transplantation (HSCT) recipients. In these subjects, the prolonged lymphopenia due to conditioning treatments or T-cell depletion is generally responsible for the often-undetectable TTV DNA. After transplantation, TTV viral load kinetics seem to follow the process of immunological reconstitution.

Therefore, the aim of this review is to evaluate TTV plasma viral load in HSCT recipients before the transplantation and during the immune reconstitution process. The results could allow for establishing a correlation between the changes in TTV DNA levels in plasma and the restoration of the immune system, consequently contributing to a better understanding of this complex interplay.

## 2. Methods

This systematic review followed the Preferred Reporting Items for Systematic Reviews and Meta-Analyses (PRISMA) 2020 guidelines (Supplementary Tables S1 and S2) [15]. The study protocol was registered in the PROSPERO International Prospective Register of Systematic Reviews (registration number: CRD420251116208).

### 2.1. Eligibility Criteria

We included randomized controlled trials (RCTs), prospective or retrospective cohort studies, case series, and cross-sectional studies published in peer-reviewed journals that reported TTV viral load kinetics in adult or pediatric patients undergoing allo-HSCT for lymphoproliferative disorders. Eligible studies were required to measure TTV viral load at baseline (pre-transplant) and during immune reconstitution (≥1 time point ≥30 days post-transplant) or at least the quantification of TTV viral load at two time points (one before and one after an episode that leads to a change in patients’ immunological activity such as opportunistic infections or GVHD occurrence). We excluded case reports; studies of SOT; HSCT studies without TTV viral load data; non-English-language publications; preprints; preclinical (in vitro or animal) studies; reviews; systematic reviews; meta-analyses; short communications; and commentaries.

### 2.2. Information Sources and Search Strategy

An electronic search was employed to find the published articles, which reported viral load kinetics of TTV in allo-HSCT recipients through the United States National Library of Medicine, PubMed (last accessed August 2025), OVID MED-LINE (last accessed August 2025), and the Cochrane Controlled Trials (August 2025). References for this review were identified with the following research term combinations: “TTV” OR “Torque teno virus” AND “HSCT” OR “immunocompromised” AND “Adults” AND “children”. No time window was applied to the search.

### 2.3. Selection and Data Collection Process

A team of two resident doctors in Infectious and Tropical Diseases of the University of Brescia, Italy, read the abstract of each scientific work and independently selected the articles according to the established criteria (MA and MS). A Professor in Infectious and Tropical Diseases of the University of Brescia, Italy (EQR) and a Professor in Microbiology of the University of Brescia (MADF) revised the included and the rejected papers. Then, the selected papers were equally distributed among each resident doctor to assess full-text eligibility. Each resident doctor read, collected, and synthesized the data for the articles assigned using a detailed database. Afterwards, the cross-checking phase was performed: each reviewer re-examined data extracted by a colleague to ensure consistency and accuracy. Disagreements were resolved by a joint discussion supervised by the Professor in Infectious and Tropical Diseases (EQR) and the Professor in Microbiology (MADF).

### 2.4. Data Items

For each included study, three independent reviewers (MA, MS, and MADF) systematically extracted data on study characteristics (first author, year, country, journal, and design) and population details, including sample size, age, gender, underlying lymphoproliferative disorder, type of graft (autologous or allogeneic), conditioning regimen, and stem cell source. Clinical and virological data were collected on the timing of sample collection, the methods used for TTV quantification, and TTV viral load expressed as log_10_ copies/mL at baseline (pre-transplant) and during immune reconstitution at the reported time points. Disagreements were resolved by a joint discussion. The main outcome of interest was the correlation between TTV load and immune reconstitution, analyzed by the absolute T-cell number. When available, the absolute T-cell number and TTV viral load were evaluated at the same time points. Furthermore, secondary outcomes were the development of opportunistic infections and/or a graft versus host diseases (GVHDs) and these data have been included if reported together with TTV viral load. Missing or unclear data were reported as “non-available” (NA). No standardized extraction tool was used. The presence of substantial clinical and methodological heterogeneity among the included studies, although assessed qualitatively during data extraction, does not allow for a quantitative synthesis. Therefore, a qualitative narrative synthesis was adopted in accordance with PRISMA guidelines to avoid misleading quantitative estimates.

### 2.5. Assessment of Risk of Bias

Two reviewers (GB and GT) used the Joanna Briggs Institute’s Critical Appraisal Checklist to assess independently the risk of bias of the included studies [16]. Risk of bias was categorized as “high” when the study reached up to 50% score “yes”; “unclear” when the study reached up 51% to 70% score “yes”; and “low” when the study reached more than 70% score “yes”. The results are represented as traffic light and weighted bar graphs generated by using the generic dataset model of the Risk of Bias Visualization (ROBVIS) package [17].

## 3. Results

### 3.1. Study Selection and Search Results

**Figure 1:**
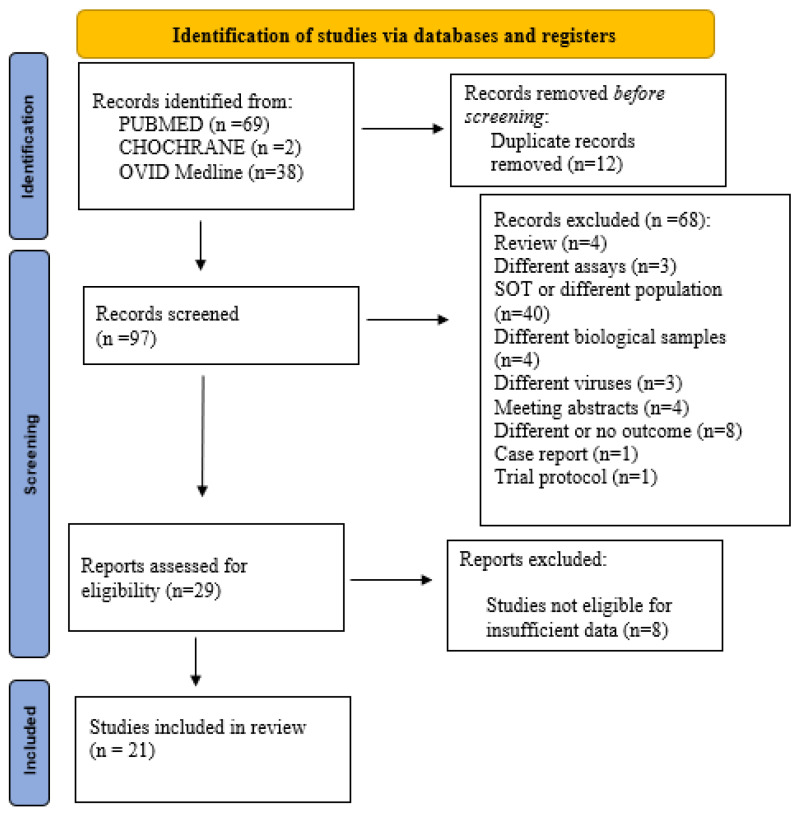
Adapted PRISMA 2020 flow diagram.

A total of 109 studies were retrieved from our search through the databases used and a total of 12 duplicate records were removed. We excluded 68 studies by screening titles and abstracts and performed a full-text review with 29 articles. Eight articles were excluded because they did not meet the inclusion criteria. A total of 21 articles were extracted for the systematic review (Figure 1).

The studies included were mostly classified as longitudinal (16/21, 76.0%) since they reported data at two or more time points of the populations analyzed, while four (19%) were retrospective studies and one was a cross-sectional study (4.7%). They were performed all in Europe (20/21, 95.2%): Spain (5/21, 23.8%), Germany (4/21, 19.0%), Italy (4/21, 19.0%), Switzerland (3/21, 14.2%), France (2/21, 9.5%), Austria (1/21, 4.7%), Belgium (1/21, 4.7%), and Turkey (1/21, 4.7%). The studies were mostly conducted on adults (19/21, 90.4%).

The study design, number of patients, and demographic and clinical characteristics of patients are summarized in Table 1.

A total of 1196 adults (median age, 54 years), males (median, 58%) were included [18,19,20,21,22,23,24,25,26,27,28,29,30,31,32,33,34,35,36]. Furthermore, a total of 76 pediatric patients, divided into two studies, were included [37,38]. Most of the adult patients received as stem cell source peripheral blood cells (713/1196, 59.6%). GVHD prophylaxis was mostly reported among the studies (13/21, 61.9%) and it was principally based on cyclosporine plus micophenolate or cyclosporine plus methotrexate.

### 3.2. Quality Assessment of the Articles

**Figure 2:**
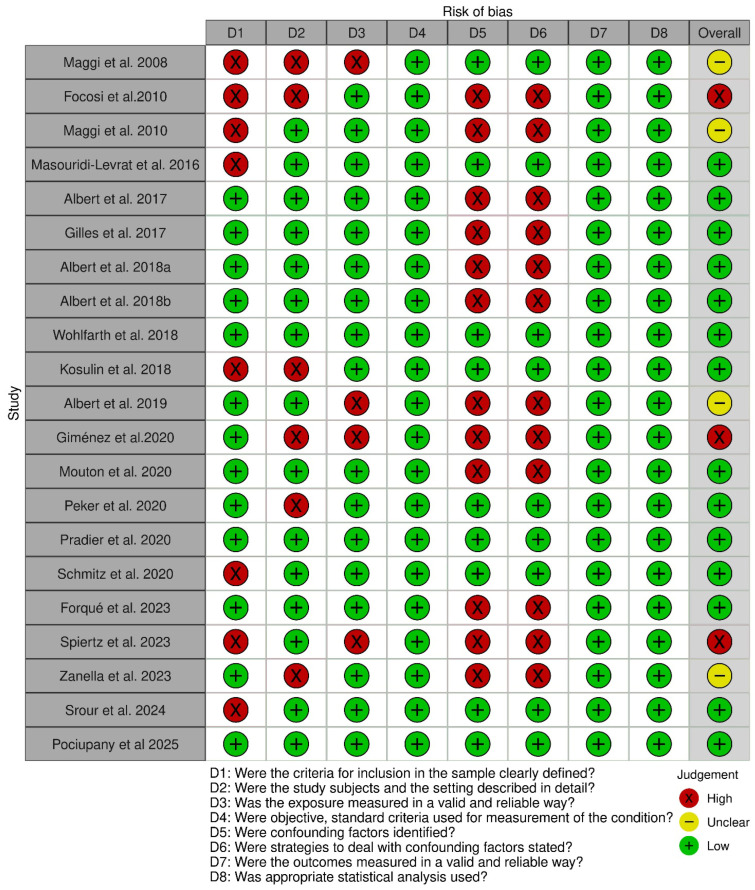
Traffic light plots of the domain-level judgements for each individual result. Maggi et al., 2008 [18]; Focosi et al., 2010 [19];Maggi et al., 2010 [20]; Masouridi-Levrat et al., 2016 [21], Albert et al., 2017 [22]; Gilles et al., 2017 [23]; Albert et al., 2018a [24]; Albert et al., 2018b [25], Wohlfarth et al., 2018 [26]; Kosulin et al., 2018 [37]; Albert et al., 2019 [27]; Giménez et al., 2020 [28]; Mouton et al., 2020 [29]; Peker et al., 2020 [38]; Pradier et al., 2020 [30]; Schmitz et al., 2020 [31]; Forqué et al., 2023 [32]; Spiertz et al., 2023 [33]; Zanella et al., 2023 [34]; Srour et al., 2024 [35]; Pociupany et al., 2025 [36].

The quality assessment of the 21 studies is summarized in Figure 2. The risk of bias analysis indicated that most of the studies included in this systematic review had a low risk of bias (67%).

However, 19% of the studies showed an unclear risk of bias and in 14% of the studies, the risk of bias was considered high. All 21 studies had well defined study questions. Some studies (8/21, 38%) did not clearly define the inclusion criteria for patients. The methods to measure TTV load were adequately described in all the studies.

**Figure 3:**
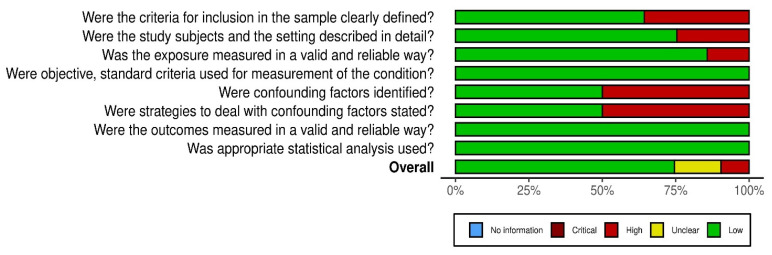
Weighted bar plots of the distribution of risk of bias judgements within each bias domain.

The principal reasons for which most of the studies have been classified as having a risk of bias were related to confounding factors and strategies to deal with confounding factors (Figure 3).

These studies included, in fact, in the analysis, very different HSCT patient groups. These patients suffered from different hematological diseases, which might have a potential different impact on their immunity, experienced different pre-transplant treatment, and also exhibit different baseline immune status—all factors that can independently affect immune recovery and TTV replication. When the authors failed to address these issues, they constituted potential confounding factors. Then, they included patients who were subjected to different prophylaxis treatment for acute graft versus host disease (aGVHD). These differences comprised different types of drugs, different doses, and different lengths of treatment. Such differences can significantly influence immune recovery and viral dynamics, introducing potential bias into the observed associations. Finally, strategies to control confounding, such as patient stratification, were often insufficient, leaving residual bias.

### 3.3. Dynamics of TTV Viral Load Before and After HSCT

All the studies reported in Table 2, 16 in adult and 2 in pediatric patients, investigated the kinetics of plasma TTV viral load after engraftment. Most of the patients tested positive for TTV before transplantation, ranging from 1 to 5.65 log_10_ copies/mL in adults and from 4 to 5.70 log_10_ copies/mL in children. A control group constituted by healthy subjects was included only in four studies [21,23,30,38]. No significant differences were observed between patient and control groups in terms of TTV DNA levels in two studies [21,38], while Peker et al. [38] found a significant difference (*p* < 0.004).

Then, Mouton et al. [29] analyzed TTV viremia in 80 healthy subjects compared to that of 41 allo-HSCT recipients after 6 months of transplant and found that TTV was detected in 68% (54/80) of healthy subjects and in all patients and that it was significantly higher in allo-HSCT recipients (3.9 vs. 2.1 log_10_ copies/mL, *p* < 0.001). Gilles et al. [23] did not report TTV viral load for patient groups before transplantation, limiting, therefore, the comparison between the two groups. Most of the included studies (52%) have the last time point of sample collection after HSCT at +90 days. Many of them [18,19,20,22,23,25,35] performed three sampling time points for evaluating TTV viremia spanning from 30 to 200 days post-transplantation. Among these studies, Albert et al. [22] did not report the median value of TTV viral load for each time point, only the median of the logarithmic increase in DNAemia. Peker et al. [38] limited the analysis of TTV viremia at early engraftment (around +20 days) and after 31–60 days. Two studies [21,28] considered only one time point after transplantation at +90 and +30 days, respectively, while others [26,30,34] performed TTV quantification at more time points until the first year after HSCT.

The method used for quantitative analysis was a quantitative real-time PCR developed in-house for all the studies except two [29,35], which used a commercial assay. Most of the tests used a highly conserved segment of the TTV untranslated region (UTR) as target, and only two studies [21,34] used an assay, which targeted the TTV open reading frame 2 (ORF2). The detection limit ranged from 10 to 100 copies/mL.

**Figure 4:**
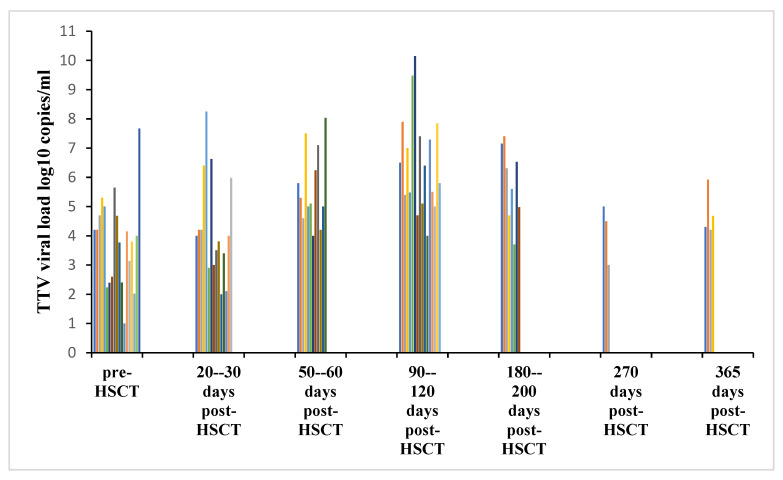
TTV DNA load kinetics in allogeneic hematopoietic stem cell transplant recipients according to the studies included in the systematic review.

Figure 4 illustrates how the results from all the analyzed studies converge with each other, indicating that TTV viral load increases after HSCT, reaching a peak mostly around days +90 and +120. In detail, Spiertz et al. [33] found that the peak of TTV viral load was reached at days +56, Peker et al. [38] at days +60, and Wohlfarth et al. [26] at days +79. Eight studies [21,22,25,27,31,32,34,35] detected the peak at days +90, and three studies [18,19,37] reported a peak at days +100, while Gilles et al. [23] found that TTV viremia peaked at days +200. After around +120 days, TTV viremia decreases, returning over one year after transplantation to basal values.

### 3.4. TTV Viral Load and Immune Reconstitution

Thirteen studies investigated whether between TTV viral load and lymphocyte numbers a correlation might be established, but the results were contradictory (Table 2). Because only a few studies reported the absolute T-cell number at the same time points of TTV viral load evaluation, we considered only their correlation. Eight studies [18,19,20,22,23,25,37,38] found that the increase in TTV DNA load over time seemed to parallel that of absolute lymphocyte numbers. Maggi et al. [18] and Focosi et al. [19] observed that the increase in TTV viremia paralleled the increase in circulating CD8+CD57+ T lymphocytes. Gilles et al. [23] reported that the increase in TTV viral load was related to a concomitant normalization of lymphocyte counts. Albert et al. [27] showed that until days +60, TTV DNAemia directly correlated with absolute lymphocyte counts (ALCs) (*p* = 0.031, rho = 0.171), while days +120 up to days +210 were inversely correlated (*p* = 0.003, rho = 0.263). According to what is proposed by the authors, TTV viral load can be considered as a biological marker for T-cell reconstitution in the early stages after HSCT, while after day +100, it might indicate a patients’ immunosuppression. In support of this hypothesis, they showed that, in patients treated with corticosteroids for treating GVHD, the median TTV DNA area under a curve between days +90 and +210 was higher than patients to whom the drug was not administered (*p* = 0.025) [27]. Kosulin et al. [37] did not find any significant correlation between the lymphocyte counts and TTV viral load at days +30, +60, or +100 post-HSCT, while they detected TTV in granulocytes where they evidenced a median DNA copy number increase of 1.8 logs between days +30 and + 60 post-transplantation in a pediatric setting. Wohlfarth et al. [26] found that, immediately afterwards the engraftment, there was an increase in TTV levels and ALCs, but then for all the subsequent follow up periods, they showed an inverse correlation (r_s_ = −0.27; *p* < 0.01). Pradier et al. [30] also found an inverse correlation between TTV viremia and the number of CD4+ T and NK cells at days +100. Furthermore, Schmitz et al. [31] did not observe any statistically significant correlation between TTV levels and different lymphocyte subpopulations such as CD3+T-cells, CD3+/CD8+ suppressor T-cells, CD3+/CD4+ T-helper cells, or CD45+ lymphocytes until day +300 of follow up. Finally, Mouton et al. [21] also detected no significant correlation between TTV viral load and ALCs or CD3+T-cells after 6 months HSCT.

### 3.5. TTV Viral Load, Opportunistic Viral Infections, and GVHD

Some studies [22,23,24,26,29,30,31,32,33,35,36] analyzed whether differences in TTV viral load might be related to the occurrence of opportunistic viral reactivation and/or of acute GVHD (aGVHD) (Table 3).

Regarding opportunistic viral infections and TTV viral load (Table 3), Gilles et al. [23] found that patients with detectable CMV, EBV, or BKPyV viremia in the first 100 days post-transplantation had higher TTV viral loads at day +30 than patients negative for these opportunistic viruses (9.26 vs. 6.40 log_10_ copies/mL, *p* = 0.005). Albert et al. [24] showed that the mean of TTV DNA load measured at the area under the curve (AUC) between 20 and 30 days post-HSCT was lower in patients with CMV viremia than in patients without it, even though it was not statistically significant (3.3 copies × days × mL^−1^ vs. 4.4 copies × day × mL^−1^). However, they found that patients with TTV DNA load AUC 20–30 < 2.8 copies × days × mL^−1^ developed high levels of CMV viremia. Then, because most of EBV reactivation episodes took place after 50 days HSCT, they investigated the TTV DNA load AUC between day 20 and day 50 after transplantation, showing that there was no significant difference between patients with and without EBV DNAemia. Wohlfarth et al. [26] reported a statistically significant correlation between TTV and CMV DNA loads and between TTV and EBV DNA loads. Then, Mouton et al. [29] found higher TTV DNA loads in patients with CMV viremia than in those who were negative for the virus (median, 4.8 vs. 3.7 log_10_ copies/mL; *p* = 0.02) about 6 months after transplantation. Schmitz et al. [31] found that virus reactivation of CMV, EBV, and BKPyV with a viral load > 1000 copies/mL, measured between 0 and 50 days after HSCT, was associated with higher TTV DNAemia, even if not statistically significant, while when the viral load was lower than 1000 copies/mL, no significant difference was observed. On the contrary, Spiertz et al. [33] showed that a TTV load < 1000 copies/mL at the early stages of HSCT was significantly (*p* not reported) related to a higher risk of CMV infection/reactivation. Pradier et al. [30] established that higher TTV titers at day 100 had higher rates of viral infections after 6 months post-transplant. Forqué et al. [32] found no significative difference in TTV viral load between patients with or without CMV DNAemia, while they observed that a DNA load cut-off ≥ 4.40 log_10_ copies/mL at pre-transplant might predict the occurrence of BKPyV-HC with a sensitivity ≥ 89%. Srour et al. [35] did not find any difference in median TTV load between the groups with and without viral infections. Finally, Pociupany et al. [36] observed a trend where higher TTV levels were present in patients with viral infections than patients without them.

Regarding the association between TTV viral load and the development of an aGVHD (Table 3), Albert et al. [22] found significant higher TTV levels in patients who developed severe aGVHD than patients without GVHD. Gilles et al. [23] observed that a TTV DNA load < 8.48 log_10_ copies/mL after 30 days HSCT combined with a lymphocyte count ≥ 5.5 × 10^8^ cells/L positively correlated with a low incidence of aGVHD within the first 100 days after allo-HSCT. Wohlfarth et al. [26] found that higher TTV DNA load in patients who had not received ATG during the conditioning was significantly associated with the development of aGVHD after days +120 and +160. Then, Pradier et al. [30], Forqué et al. [32], and Srour et al. [35] showed that patients with high TTV DNA loads after 100, 30, and 60 days, respectively, had a significantly higher risk of developing aGVHD. Finally, Schmitz et al. [31] did not observe any significant difference in TTV DNAemia between groups of patients with and without aGVHD (Table 3).

## 4. Discussion

The hypothesis that TTV, one of the first viruses recognized as part of the human virome, might be used to assess the function of the immune system has developed quickly in recent years. TTV, in fact, possesses several notable features that make it a reliable immune marker [39], such as its widespread prevalence, extensive distribution, resistance to antiviral treatments, and the possibility to measure DNA with different PCR assays. However, while TTV viremia could be considered as an indirect measure of immune function in SOT recipients as confirmed by different studies [2,40,41,42,43,44,45,46,47], the clinical utility of TTV viral load in HSCT patients is still under debate, underlining the profound biological differences between these two transplant modalities.

For this reason, our systematic review analyzed whether TTV might be considered a biological marker of immune competence also in the HSCT setting.

The results showed that there was a consensus between all the studies included about the kinetics of TTV DNA viral load, which followed a characteristic pattern. It, in fact, decreased dramatically after conditioning therapy and reached the lowest levels around the time of hematopoietic engraftment; then, the TTV viremia steadily increased, reaching peak levels at day +90 and +120 in most of the analyzed studies. After about 100 days, TTV viral load slightly decreased until it reached a stable plateau over one year after transplantation.

Because hematopoietic cells are considered the principal competent cells for TTV replication, it was suggested that the increase in TTV viral load in blood compartment parallels the repopulation of lymphocytes and therefore measuring the TTV levels might be useful to assess both the hematological and immunological reconstitution.

The results provided by the studies were conflicting. Six studies [18,19,20,22,23,25], performed in adult patients and monitored for about 100 days after HSCT, found that the increase in TTV DNA load over time seemed to parallel that of ALCs. However, this correlation can be explained by the ALC reconstitution post-engraftment, which then serves as a TTV replication reservoir, but did not allow for extrapolating a correlation between TTV and T-cell function.

Three studies [26,27,30], on the contrary, found an inverse correlation between ALCs and TTV viral load after 100 days post-transplantation.

In pediatric patients, Kosulin et al. [37] evidenced no significant correlation between the ALCs and TTV levels at +30, +60, or +100 days post-transplant in 43 children. However, they found that the virus replicated in CD15+ cells, the most representative fraction of neutrophilic granulocytes, revealing that TTV levels correlated significantly with the number of neutrophils at +30 and +60 days after HSCT. Furthermore, they found that expansion of the virus generally starts 1 month after transplantation, with the engraftment of these cells. This finding might suggest that high granulocyte numbers are needed for efficient TTV replication post-transplant and could provide a reason for the delayed initiation of rapid replication of the virus. Then, Peker et al. [38] found in 33 pediatric patients that lymphocyte counts and TTV viral load were positively correlated even if weakly (Spearman’s rank test, rho = 0.29, *p* = 0.001). These two studies provided additional information about the potential impact of TTV viral load in pediatric HSCTs where data are not available except for a paper that describes a correlation between the chemokine MCP-3 and TTV positivity [48].

Furthermore, in two studies [37,38], higher TTV viral loads were detected after transplantation compared to those observed in adults. This difference could be ascribed to new infections with different genotypes, as detected in a longitudinal study in children [9]. The diversity of TTV genotypes has been found, in fact, to increase in transplant recipients because of engraftment and the transfusion of blood products from multiple donors during the post-transplant period [19].

Based on these results, TTV dynamics might exhibit Janus-like behavior, playing two opposite roles: in the early stages after HSCT, it might parallel immunological reconstitution, and in the long-term after transplantation (around +100 days), TTV viral load might represent the degree of the patient’s immunosuppression.

However, establishing which cut-off value for TTV viral load should be used as watershed between immunosuppression and immunocompetence remains an open question.

TTV-related immune dynamics in patients with hematologic disorders are very complex because immune reconstitution is a multifaceted process that varies significantly between individuals and includes both quantitative and functional parameters of ALC [49].

Therefore, variability found among studies may be influenced by many factors such as the source of the graft (BM, PB, and CB stem cells), which affects immune cell reconstitution. It was, in fact, found that cord blood stem cells led to earlier NK/B reconstitution than BM or PB, skewing TTV kinetics [50]. Another source of variability is linked to the conditioning regimen administered prior to transplantation and to the use of T-cell depletion (e.g., ATG, alemtuzumab), which profoundly affects immune recovery kinetics and TTV replication. Both myeloablative conditioning and higher ATG doses were found to correlate with elevated TTV titers [31,35].

These interventions can delay lymphocyte reconstitution and confound the relationship between TTV load and immune status.

Furthermore, other influencing factors might be due to the cell dose, human leukocyte antigen compatibility, and post-transplant interventions aimed at preventing or managing complications such as opportunistic infections and severe GVHD [51]. Viral interactions are complex and not completely studied; therefore, differences in antiviral prophylaxis (drug type, dose, duration) influence both viral replication and immune dynamics. Herpesviruses, for example, can induce the amplification of other persisting viral DNA. Higher levels of TTV replication have indeed been described in EBV-infected cell lines [52] and persistent human cytomegalovirus infection has been significantly related to higher TTV DNA loads, especially at a younger age [53].

Furthermore, the observed differences might be assigned to different time points chosen for follow up because TTV levels fluctuate during the post-transplant period, and some time point measurements may not capture meaningful trends in immune recovery. Then, another limiting factor is the genomic variability of TTV. Due to the extensive sequence diversity, even within its most conserved region, the efficiency of qPCR amplification can vary across different TTV species, potentially leading to inaccuracies, which might limit its clinical applicability [54].

Then, variability in PCR targets (UTR or ORF 2 regions) across studies can also lead to differences in amplification efficiency and quantification accuracy, making inter-study comparisons problematic.

Similarly, some studies analyzed a possible association between TTV-DNA levels and episodes of viral opportunistic infections and GVHD, but they again yielded inconsistent results.

The discrepancies in findings may stem from heterogeneity in study designs. Some studies focus on correlation, others on predictive value, leading to inconsistent conclusions. Then, lack of standardized monitoring windows reduces the ability to establish causality or predictive accuracy.

Furthermore, post-transplant immunosuppressive therapy is highly variable, influencing significantly both TTV replication and the risk of opportunistic infections or GVHD. Agents such as calcineurin inhibitors (e.g., cyclosporine, tacrolimus) and high doses of corticosteroids can suppress T-cell function to different degrees and durations, with a significant effect on viral control mechanisms. Stronger or prolonged immunosuppression may allow for the persistence of higher TTV loads, which therefore are not necessarily correlated to poor immune recovery but rather to pharmacologic suppression. Future studies should analyze how specific agents influence TTV kinetics and immune cell subsets, while integrated approaches combining TTV load with functional immune assays could improve specificity.

Furthermore, in general, even if a low-risk bias assessment was classified in most studies (67%), it was possible to identify important clinical and methodological differences.

In fact, substantial variability remains in their design, ranging from cross-sectional and retrospective to longitudinal approaches. Additional inconsistencies include imbalances in baseline characteristics, small sample sizes with more than half (52%) of the studies evaluating fewer than 50 patients, differences in age groups (adults vs. children), diagnostic techniques, immune parameters assessed, and the inclusion or absence of control groups. Finally, publication bias cannot be excluded, although an assessment using funnel plots or statistical tests was not performed due to outcome heterogeneity. So far, there is the possibility that negative or inconclusive results were underreported, potentially affecting the overall interpretation of evidence.

## 5. Conclusions

TTV plasma viral load may reflect the degree of immunosuppression and immune reconstitution after HSCT and therefore could serve as a candidate biomarker in patients with immunoproliferative disorders, although current evidence remains heterogenous.

**Figure 5:**
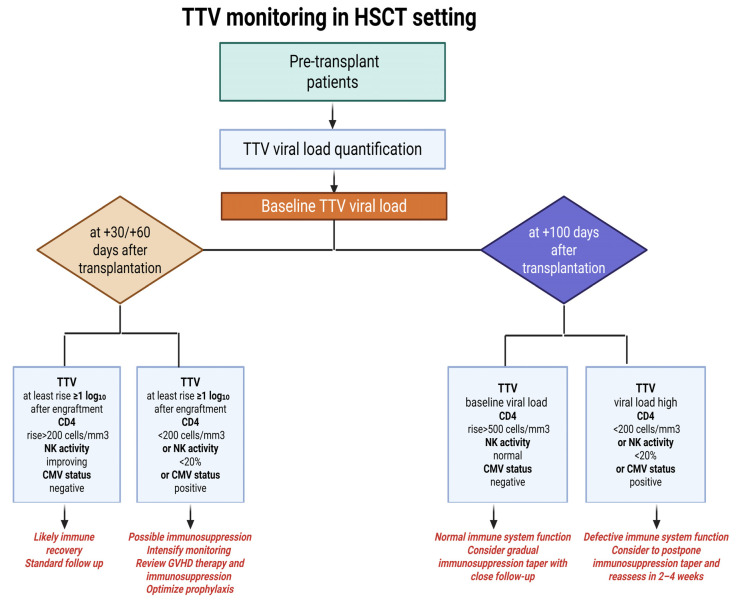
TTV-guided immune monitoring after HSCT. Patients are sampled at standardized time points (pre-HSCT, at days +30, +60, +100). At each time point, TTV viral load (qPCR) detection should be integrated with CD4+ T-cell counts, NK cell activity, and CMV DNAemia. Suggested actions include intensified monitoring or adjustment of immunosuppression when risk markers are high, versus cautious taper when the composite profile indicates recovery. Created by using Biorender.com.

Nevertheless, evidence from the analyzed studies suggests that during the early post-transplant phase (approximately day +30 to +60), increasing TTV levels may parallel immune recovery, in terms of lymphocyte reconstitution. Conversely, in the later phase (>100 days), persistently high TTV loads are more likely to indicate ongoing immunosuppression, particularly in patients receiving intensive GVHD prophylaxis or treatment. To implement TTV monitoring in routine practice, standardized sampling at key time points (baseline, day +30, +60, and +100) should be combined with complementary functional markers such as CD4+ T-cell counts, NK cell activity, and CMV reactivation to guide immunosuppressive treatment and infection risk stratification, as proposed in Figure 5.

Future studies and standardized protocols are essential to establish clinically meaningful cut-off values that are able to clarify whether TTV viral load evaluated at specific time points reflects immune recovery or immunosuppression.

## Figures and Tables

**Table 1:** Summary of characteristics of included studies and patients.

Source	Year	Country	Study Design	Patient Number	Male, *n* (%)	Patient Age, Median (Range)	Clinical Endpoint	Donors (*n*)	Stem Cell Source (*n*)	Diagnosis (*n*)	Conditioning	Prophylaxis (*n*)	Reference
Maggi et al.	2008	Italy	Longitudinal	19	NA	NA	IR	NA	PB (15)	MM (19)	MAC	NA	[18]
Focosi et al.	2010	Italy	Longitudinal	47	NA	NA	IR	Related	PB (47)	MM (36), LY (10), AML (1)	MAC (37), HCDT (10)	NA	[19]
Maggi et al.	2010	Italy	Longitudinal	4	1 (25)	50 (41–58)	IR	NA	PB (4)	T-ALL (1), B-ALL (1), ALL (1), AML (1)	NA	NA	[20]
Masouridi-Levrat et al.	2016	Switzerland	Longitudinal	121	67 (55)	50 (18–70)	IR	MMURD (10), MMRD (7), MSD (29), MUD (37)	PB (121)	AML (58), ALL (15), MDS (12), NHL (10), MPS (6), MM (9), HL (5), CML (39, CLL (1), MDPS (1)	NA	NA	[21]
Albert et al.	2017	Spain	Longitudinal	72	38 (53)	54 (18–69)	aGVHD	Related (37); unrelated (35); matched (49); mismatched (23)	PB (68); BM (3); CB (1)	HL (5); NHL (15); CLL (6); ALL (7); AML (19); CML (1); MM (5); MDS (10); others (4)	MAC (13); NMA (59)	Steroids (45), ASCT (30), TKI (16)	[22]
Gilles et al.	2017	Germany	Longitudinal	23	13 (57)	57 (33–75)	IR	MRD (5); MUD (8); MMURD (9); haploidentical (1)	PB (23)	AML (11); ALL (1); CLL (1); CMML (1); others (9)	RIC (23)	CSA/MMF (18), CSA/MTX (2), ATG/CSA (1), CSA/TAC (1), MMF/TAC (1)	[23]
Albert et al.	2018a	Spain	Longitudinal	71	42 (59)	55 (18–70)	OI	Related (39); unrelated (32); matched (50); mismatched (21)	PB (69); BM (1); CB (1)	HL (3); NHL (21); CLL (6); ALL (6); AML (15); MM (5); MDS (7); others (8)	MAC (13), NMA (58)	CSA or MTF/TAC (24), CSA or MMF/TAC (29), regimens including thymoglobulin (4), regimens including cyclophosphamide (14)	[24]
Albert et al.	2018b	Spain	Longitudinal	38	22 (58)	55 (26–69)	IR	Related (22); unrelated (16); matched (26); mismatched (12)	PB (37), BM (1)	HL (1), NHL (14), ALL (4), CLL (3), AML (6), MM (2), MDS (6), others (2)	MAC (5); RIC (33)	CSA or MTF/TAC (13), CSA or MMF/TAC (18), regimens including cyclophosphamide (7)	[25]
Wohlfarth et al.	2018	Austria	Longitudinal	50	32 (64)	49 (37–57)	IR, OI, aGVHD	Unrelated (35), SIB (13), mismatched (18)	PB (45), CB (3), BM (2)	AML (25), ALL (9), MDS (6), NHL (3), others (7)	MAC (26), RIC (16), NMA (8)	CSA + MTX (26), CSA + MMF (24)	[26]
Albert et al.	2019	Germany	Longitudinal	33	16 (48)	56 (19–70)	IR	Related (18), unrelated (15)	PB (31), BM (1), CB (1)	Lymphoma (12), leukemia (12), myeloma (4), MDS (3), MF (2)	MAC (6), NMA (27)	CSA + MTX or MMF (19), TAC + SIRO (14)	[27]
Gimenez et al.	2020	Spain	Longitudinal	25	14 (56)	54 (24–69)	IR	MRD (7), MUD (8), MMRD (5), Haplo (5)	PB (23), BM (2)	Lymphoma (10), AML (4), ALL (4), MDS (2), CLL (3), MM (2)	NA	NA	[28]
Mouton et al.	2020	France	Cross-sectional	41	25 (61)	56 (40–64)	IR, OI	MRD (23), MUD (15), MMURD (3)	PB (28), BM (13)	Myeloid neoplasm and acute leukemia (37), others (4)	MAC (17), RIC (24)	100% but not reported specifically for each patient	[29]
Pradier et al.	2020	Switzerland	Longitudinal	168	104 (62)	51 (39–59)	IR, aGVHD	SIB (71), MUD (75), MMUD (13), Haplo (9)	PB (149), BM (19)	AML (78), ALL (17), MDS (22), MPS (11), lymphoma (12), myeloma (11)	RIC (85), MAC (83)	CSA + MMF (85), CSA + MTX (83)	[30]
Schmitz et al.	2020	Germany	Retrospective	123	74 (60)	54 (19–75)	IR, OI, aGVHD	Unrelated (90), related (33), matched (105), mismatched (18)	PB (116), BM (7)	AML (58), ALL (9), MDS (33), NHL (11), others (12)	MAC (48), RIC (75)	MMF + CSA (30), MMF + TAC (83)	[31]
Forqué et al.	2023	Spain	Retrospective	75	43 (57)	54 (19–70)	IR,OI, aGVHD	Related (46), unrelated (29), matched (41), mismatched (9), Haplo (25)	PB (75)	AML (28), ALL (1), CML (3), CLL (3), HL (13), MM (1), MDS (4), MF (4), NHL (15), others (3)	MAC (14), RIC (61)	SIRO + MMF + cyclophosphamide (71), TAC + SIRO (2), TAC + CP (2)	[32]
Spiertz et al.	2023	Germany	Retrospective	59	33 (56)	52 (19–74)	IR,OI	MUD (36), MRD (13), MMUD (7), MMRD haplo-identical (3)	NA	AML (31), ALL (6), CML (2), CLL (4), MDS (10), others (6)	RIC (54), MAC (5)	CSA + MMF or MTX (59)	[33]
Zanella et al.	2023	Switzerland	Longitudinal	109	72 (66)	56 (no range)	IR	MUD (47), MSD (28), Haplo (24), MMURD (10)	PB (96), BM (13)	AML (59), MDS/MDPS (27), ALL (10), MPS (4), lymphoma (4), CLL (2), myeloma (2), CML (1)	RIC (74), MAC (43)	NA	[34]
Srour et al.	2024	France	Longitudinal	70	41 (59)	54 (19–73)	IR, OI, aGVHD	MRD (14), MUD (40), MMURD (5), Haplo (11)	PB (37), BM (33)	HL (1), NHL (6), MM (2), ALL (13), AML (27), CML (1), MDS (13), MF (3), others (4)	MAC (36), RIC (34)	NA	[35]
Pociupany et al.	2025	Belgium	Longitudinal	48	28 (58)	PTLD patients 52 (16–67), not PTLD 53 (0–68)	OI	MUD (27), MRD (15), Haplo (6), CB (1)	PB (40), BM (8)	HL (3), T-cell/NK-cell lymphoma (3), MCL (1), ALL (3), AML (18), MDS (5), MF (1), CML (1), MM (1), others (5), NA (7)	MAC (29), NMA (29)	ATG, CSA + MTX (7), ATG, CSA + MMF (1), CSA + MMF (4), MMF (1), CSA (2), CSA + CP (3), others (3), NA (27)	[36]
Kosulin et al.	2018	Italy	Longitudinal	43	NA	Pediatric patients (age NA)	IR	Unrelated (27), MSD (10), MMRD (6)	NA	HM (45), SCID (7), FAA (1)	MAC (22), RIC (21)	NA	[37]
Peker et al.	2020	Turkey	Retrospective	33	19 (58)	7.8 (0.7–18.6)	IR	MUD (19), MRD (11), autolog (2), Haplo (1)	PB (16), BM (16), CB + BM (1)	HLH (2), AML (7), ALL (5), TM (8), FAA (3), JMML (1), WAS (1), SCID (2), CN (2), NB (1)	MAC (21), NMA (12)	CSA + MTX (18), CSA + MMF (4), CSA (6), CSA + MMF + MTX (3), NA (2)	[38]

ALL, acute lymphoid leukemia; AML, acute myeloid leukemia; ASCT, autologous stem cell transplantation; ATG, anti-thymocyte globulin; B-ALL, B-cell acute lymphoblastic leukemia; BM, bone marrow; CB, cord blood; CLL, chronic lymphoid leukemia; CML, chronic myeloid leukemia; CMML, chronic myelomonocytic leukemia; CN, Congenital Neutropenia; CP, cyclophosphamide; CSA, ciclosporin; FAA; Fanconi aplastic anemia; aGVHD, acute graft versus host disease; Haplo, haploidentical donor; HL, Hodgkin lymphoma; HLH, hemophagocytic lymphohistiocytosis; HM, hematological malignancies; IR, immune reconstitution; JMML, juvenile myelomonocytic leukemia; MAC, myeloablative conditioning; MDPS, myelodysplastic/myeloproliferative syndrome; MCL, Mantle cell lymphoma; MDS, myelodysplastic syndrome; MF, myelofibrosis; MM, multiple myeloma; MMF, mycophenolate mofetil; MMRD, mismatched related donor; MMURD, mismatched unrelated donor; MPS, myeloproliferative syndrome; MRD, matched related donor; MSD, matched sibling donor; MTX, methotrexate; MUD, matched unrelated donor; NA, not available; NB, neuroblastoma NHL, non-Hodgkin lymphoma; NMA, non-myeloablative conditioning; OI, opportunistic infections; PB, peripheral blood stem; PTLD, post-transplant lymphoproliferative disorder; RIC, reduced intensity conditioning; SCID, severe combined immunodeficiencies; SIRO, sirolimus; TAC, tacrolimus; T-ALL, T-cell acute lymphoblastic leukemia; TKI, tyrosine kinase inhibitor; TM, thalassemia major; WAS, Wiskott Aldrich syndrome.

**Table 2:** TTV viral load changes at pre- and post-transplantation and correlation with absolute T-cell number.

First Author, Year of Publication	Number of Patients Included in Follow Up	Control Group	Quantitative Method Used, Target Gene	TTV DNAemia * Pre-HSCT	TTV DNAemia * Post-HSCT (20–30 Days)	TTV DNAemia * Post-HSCT (50–60 Days)	TTV DNAemia * Post-HSCT (90–120 Days)	TTV DNAemia * Post-HSCT (180–200 Days)	TTV DNAemia * Post-HSCT (270 Days)	TTV DNAemia * Post-HSCT (365 Days)	ALCs in Correlation with TTV Load
Maggi, 2008 [18]	19	No	RT PCR in-house, UTR, detection limit = NR	4.2	4	5.8	6.5	NA	NA	NA	Direct correlation (r = 0.049, *p* = 0.001)
Focosi, 2010 [19]	47	No	RT-PCR in-house, UTR detection limit = 100 copies/ mL	4.2	4.2	5.3	7.9	NA	NA	NA	Direct correlation (r = 0.062, *p* = 0.001)
Maggi, 2010 [20]	3	No	RT-PCR in-house, UTR detection limit = 100 copies/ mL	Pz n°1 = 4.7 Pz n°2 = 5.3 Pz n°3 = 5.0	Pz n°2 = 4.2	NA	Pz n°1 = 5.4 + 80 d Pz n°3 = 7.0 + 110 d	NA	NA	NA	Direct correlation (r not determined)
Masouridi-Levrat, 2016 [21]	77	Yes 74 HS	RT-PCR in-house, ORF2 detection limit = 25 copies/mL	HS = 2.23 Pz = 2.39	NA	NA	5.48	NA	NA	NA	Not determined
Albert, 2017 [22]	55	No	RT-PCR in-house, UTR detection limit = 10 copies/mL	TTV DNA loads ranging from 1.40 to 7.97 in 32 Pz	After 30 days a median increase of 3.34 in 16 Pz	TTV DNA load continued to rise, with a median increase of 4.43 in 22 Pz.	TTV DNA load peaked, with a median increase of 5.02 in 19 Pz	NA	NA	NA	Direct correlation (r = 0.285, *p* = 0.032)
Gilles, 2017 [23]	23	Yes 16 HS	RT-PCR in-house, UTR detection limit = 100 copies/mL	HS BL = 5.08 LR Pz = 12 HR Pz = 11 TTV viral load baseline for Pz was not reported	8 LR Pz = 6.40 11 HR Pz = 9.26	NA	10 LR Pz = 9.48 9 HR Pz = 10.15	12 LR Pz = 7.15 11 HR Pz = 7.40	NA	NA	Direct correlation (r not determined)
Albert, 2018b [25]	Pre-transplant = 23 FU at +30 = 24 FU at +50 = 32 FU at +90 = 25	No	RT-PCR in-house, UTR detection limit = 10 copies/mL	2.6 = 20 Pz pos/23	2.9 = 16 Pz pos/24	4.6 = 30 Pz pos/32	4.7 = 24 Pz pos/25	NA	NA	NA	Direct correlation (r = 0.317, *p* = 0.002)
Wohlfarth, 2018 [26]	40	No	RT-PCR in-house, UTR detection limit = 10 copies/mL	5.65	6.63	7.50	7.40	6.31	5.0	4.68	Inverse correlation (r = −0.27, *p* < 0.01)
Mouton, 2020 [29]	41	Yes 80 HS	TTV R-GENE^®^ kit (BioMérieux, Marcy-l’Étoile, France ) (bioMérieux) Detection limit = 10 copies/mL	NA	NA	NA	NA	3.1 = 41 Pz 2.1 = 54/80 HS	NA	NA	No correlation (r = −0.13, *p* = 0.42)
Albert, 2019 [27]	33	No	RT PCR in-house, UTR, detection limit = 10 copies/mL	4.68	3 after 20 d 3.5 after 30 d	5	5.1	4.7	4.5	NA	Direct correlation until +60 days (r = 0.171, *p* = 0.031), then inverse correlation (r = −0.263, *p* = 0.003)
Giménez, 2020 [28]	25	No	RT-PCR in-house, UTR detection limit = 10 copies/mL	3.77	3.81	NA	NA	NA	NA	NA	Not determined
Pradier, 2020 [30]	130	Yes 91 HS	RT-PCR in-house, UTR detection limit = 25 copies/mL	2.2 = 91 HS 2.4 = 130 Pz	NA	5.1 = 124 Pz	6.4 = 115 Pz	5.6 = 95 Pz	4.7 = 81 Pz	4.3 = 64 Pz	Inverse correlation at day 100 (r = −0.271, *p* = 0.005)
Schmitz, 2020 [31]	123	No	RT-PCR in-house, UTR detection limit = 100 copies/mL	1 = 62 Pz	~2 §	~4 §	~4 §	~3.7 §	~3 §	NA	No correlation (r = 0.092, *p* not indicated)
Forqué, 2023 [32]	75	No	RT PCR in-house, UTR, detection limit = 10 copies/mL	4.15 = 52 Pz	3.40 = 64 Pz	6.24 = 65 Pz	7.29 = 61 Pz	6.53 = 33 Pz	NA	NA	Not determined
Spiertz, 2023 [33]	59	No	RT PCR in-house, UTR, detection limit = 100 copies/mL	3.14	NA	7.10	NA	NA	NA	5.92	Not determined
Zanella, 2023 [34]	109	No	RT-PCR in-house, ORF2 Detection limit = 25 copies/mL	3.8 = 42 Pz	NA	4,2	5.51 = 93 Pz	4.98 = 79 Pz	NA	4.2 = 48 Pz	Not determined
Srour, 2023 [35]	70	No	TTV R-GENE^®^ kit (bioMérieux) Detection limit = 10 copies/ mL	2.02	2.1	5	5.8 after 90 d 5 after 120 d	NA	NA	NA	Not determined
Kosulin, 2018 [37]	43 pediatric patients	No	RT-PCR in-house, UTR detection limit = 10 copies/mL	4	4	NA	7.84	NA	NA	NA	Direct correlation with granulocytes at +30 and +60 days (r = 0.494, *p* = 0.002)
Peker, 2020 [38]	33 pediatric patients	Yes 38 HS	RT-PCR in-house, UTR detection limit = 100 copies/mL	5.51 = HS 7.67 = Pz	5.98 + 20 d	8.03	NA	NA	NA	NA	Direct correlation (r = 0.29, *p* = 0.001)

* Expressed as mean [log_10_ copies/mL]; §, extrapolated approximately from the figure; ALCs, absolute lymphocyte counts; d, days; HSCT, hematopoietic stem cell transplantation; HS, healthy subjects; ORF, open reading frame; NA, not available; NR, not reported; Pz, patient; UTR, untranslated region.

**Table 3:** TTV viral load in relation to viral opportunistic infection reactivation and/or the development of an aGVHD after HSCT.

First Author, Year of Publication	TTV Viral Load * Post-HSCT in Patients Without OI	TTV Viral Load * Post-HSCT in Patients with OI	*p*	TTV Viral Load * Post-HSCT in Patients Without aGVHD	TTV Viral Load * Post-HSCT in Patients with aGVHD	*p*
Albert, 2017 [22]	Not determined	Not determined	NA	4 after 60 days	4.8 after 60 days	**0.09**
Gilles, 2017 [23]	6.40 after 30 days	9.26 after 30 days	0.005	<8.48 after 30 days was related to lower incidence of aGVHD		NA
Albert, 2018a [24]	4.4 for CMV risk AUCs20–30 7.10 for EBV risk AUCs20–50	3.3 for CMV risk AUCs20–30 6.67 for EBV risk AUCs20–50	0.123 0.31	Not determined	Not determined	NA
Wohlfarth, 2018 [26]	4.28 after 365 days	5.40 after 365 days	**0.04**	5.76 after 120 days 5.29 after 160 days	7.59 after 120 days 6.96 after 160 days	**0.04** **0.01**
Mouton, 2020 [29]	3.2 for OI after 6 months 3.7 for CMV risk after 6 months	4.1 for OI after 6 months 4.8 for CMV after 6 months	**0.02** **0.02**	Not determined	Not determined	NA
Pradier, 2020 [30]	Higher TTV titers at day 100 had higher rates of infection		NA	5.6 after 100 days	6.9 after 100 days	**0.013**
Schmitz, 2020 [31]	Virus reactivation > 1000 copies/mL was associated with a higher but not significant TTV viral load		>0.05	The group of patients without GVHD vs. patients with an aGVHD of grade II or higher revealed no significant difference in TTV viral load		>0.05
Forqué, 2023 [32]	3.14 after 30 days	3.80 after 30 days	0.46	2.66 after 30 days	4.53 after 30 days	**0.02**
Spiertz, 2023 [33]	3.03 at early stage of HSCT	4.40 at early stage of HSCT	NA	Not determined	Not determined	NA
Srour, 2023 [35]	No difference in TTV viral load between the two groups of patients		NA	4.97 after 60 days	6.18 after 60 days	**0.02**
Pociupany, 2025 [36]	4.94 after 90 days	7.56 after 90 days	0.059	Not determined	Not determined	NA

* Expressed as mean [log_10_ copies/mL]; OI, opportunistic infections; aGVHD, acute graft versus host disease; HSCT, hematopoietic stem cell transplantation; AUC, area under the curve; NA, not available; *p* values statistically significant are in bold.

## Data Availability

Not applicable.

## Supplementary Materials

The following supporting information can be downloaded at: https://www.mdpi.com/article/10.3390/pathogens15010105/s1, Table S1: PRISMA 2020 abstract checklist; Table S2: PRISMA 2020 27-item checklist.
